# Supplementary material for: Sulfur-enriched sub-arc fluids drive deep sulfur cycling in subduction zones
Source: Nat Commun. 2026 Apr 7;17:4953. doi: 10.1038/s41467-026-71439-3 (PMC13234425; doi:10.1038/s41467-026-71439-3)
Supplement: Supplementary file 1 — Supplementary Information [file 41467_2026_71439_MOESM1_ESM.pdf]

Supplementary Information for

Sulfur-enriched sub-arc fluids drive deep sulfur cycling in subduction zones

Dong-Bo Tan[1], Yilin Xiao[1,2,*], Yibing Li[3], Haiyang Liu[4], Deshi Jin[1], Yang-Yang Wang[1], Xiaoguang Li[5], Haihao Guo[6], Zeng-Li Guo[7], Carlos J. Garrido[8], Timothy Kusky[6]

[1]State Key Laboratory of Lithospheric and Environmental Coevolution, School of Earth and Space Sciences, University of Science and Technology of China, Hefei 230026, China
[2]Chinese Academy of Sciences Center of Excellence in Comparative Planetology, Hefei 233026, China
[3]Marine Science and Technology College, Zhejiang Ocean University, Zhoushan 316022, China
[4]Key Laboratory of Ocean Observation and Forecasting, Center of Deep Sea Research, Chinese Academy of Sciences, Qingdao, China
[5]State Key Laboratory of Lithospheric and Environmental Coevolution, Institute of Geology and Geophysics, Chinese Academy of Sciences, Beijing, China
[6]State Key Laboratory of Geological Processes and Mineral Resources, China University of Geosciences, School of Earth and Planetary Sciences, Wuhan 430074, China
[7]SK Lab-DeepMinE, MOE KLab-OBCE, School of Earth and Space Sciences, Peking University, Beijing 100871, China
[8]Instituto Andaluz de Ciencias de la Tierra (IACT–CSIC), CSIC, Granada, Spain

Corresponding author. Email: ylxiao@ustc.edu.cn

This content file includes:

Figs. S1 to S7

Supplementary Data 1–5 (provided as separate Excel files)

Figures

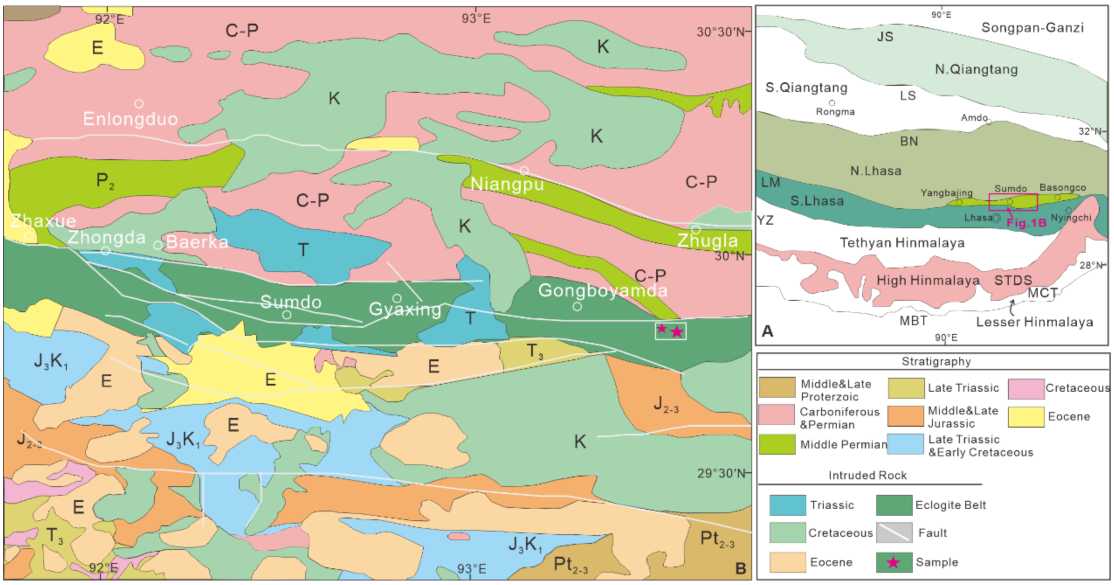

Fig. S1 Tectonic context of the Sumdo eclogite belt. (A) Simplified geological map of the Tibetan Plateau. (B) Regional geological map of the Sumdo eclogite belt within the eastern Lhasa terrane. The Sumdo belt represents a fossil subduction zone associated with Paleo-Tethyan oceanic convergence. Modified after Liu et al[1].

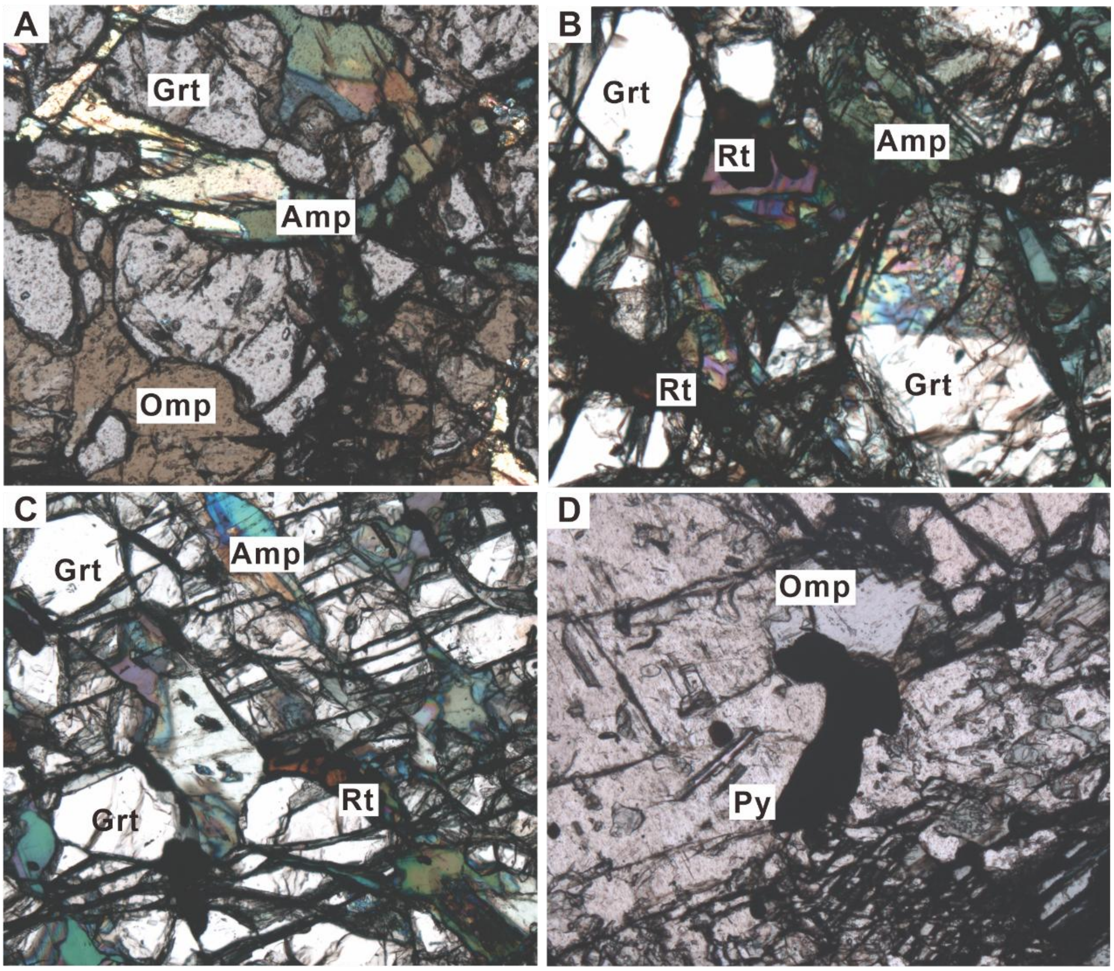

Fig. S2 Representative photomicrographs illustrating the mineral assemblages of the Sumdo eclogites. The elogites are composed of omphacite (Omp), garnet (Grt), rutile (Rt), amphibole (Amp), and pyrite (Py).

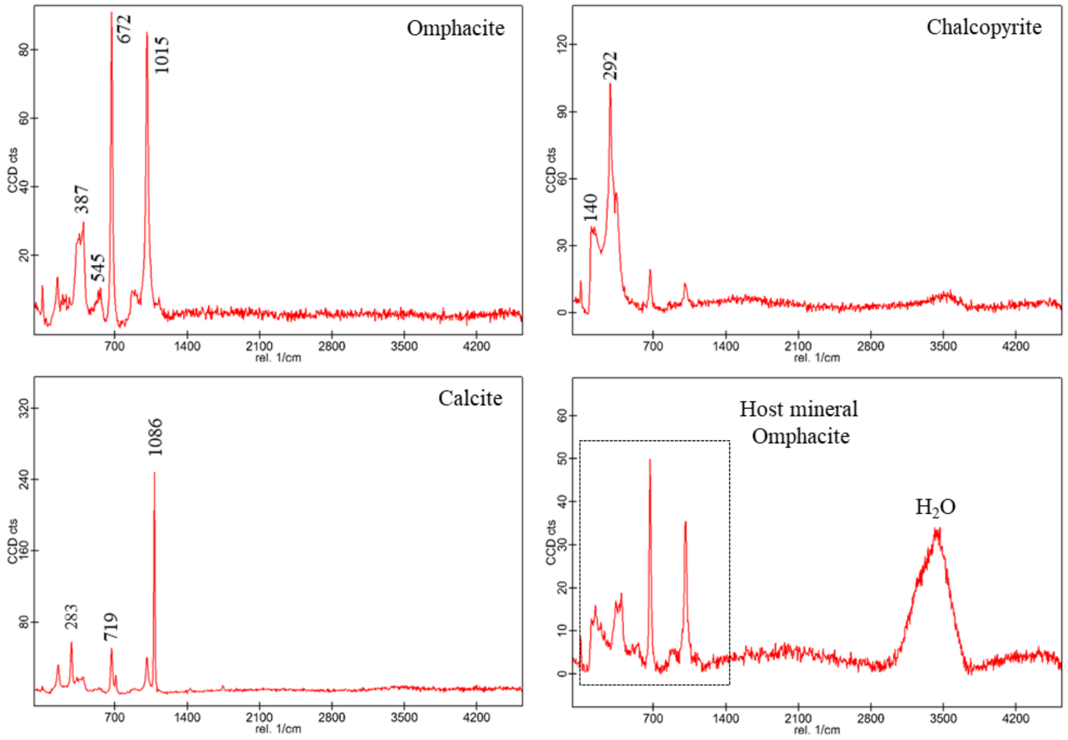

Fig. S3 Representative Raman spectra of daughter minerals and fluid phases in multiphase fluid inclusions. Raman spectra of chalcopyrite, calcite, and aqueous fluid phases within multiphase inclusions hosted in omphacite from the Sumdo eclogite. Details of mineral identification and discrimination from compositionally similar phases are provided in the Methods.

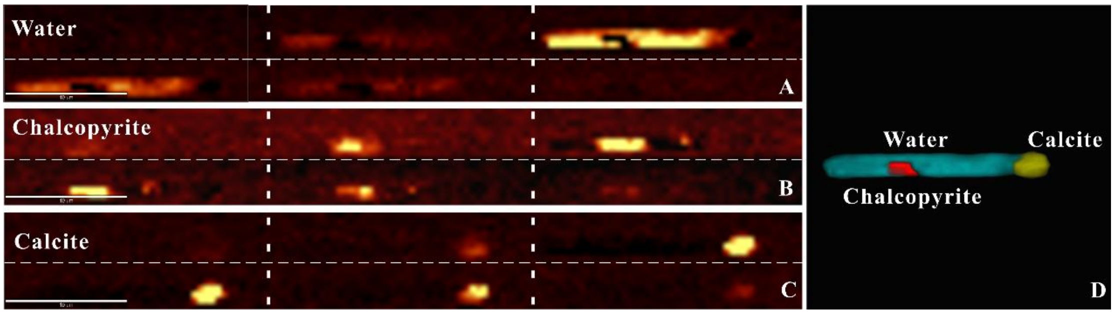

Fig. S4 Schematic illustration of 3D modeling of a multiphase fluid inclusion. (A–C) Sequential Raman depth slices (I-VI) showing the distribution of H_2O, chalcopyrite, and calcite from the surface to the interior of the inclusion. (D) Reconstructed 3D model depicting the spatial distribution of individual phases within the fluid inclusion.

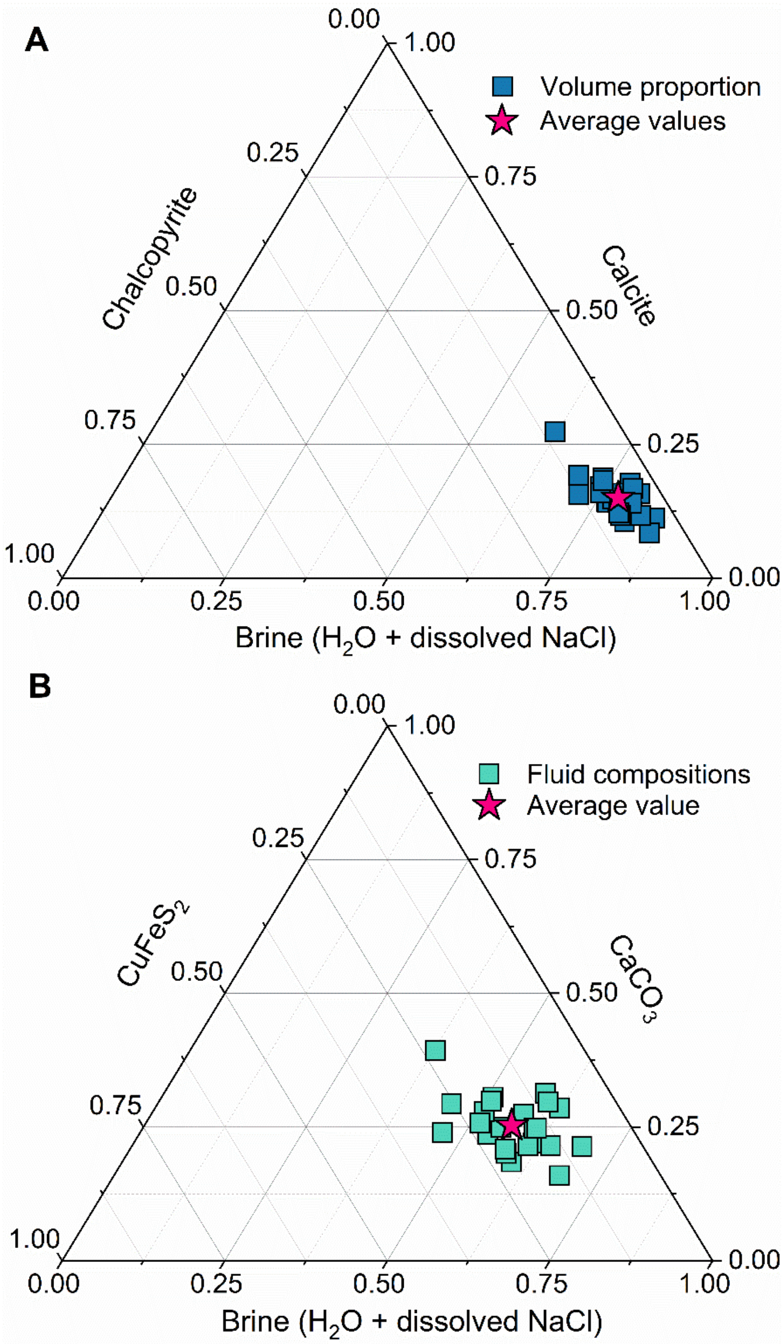

Fig. S5 Phase proportions and reconstructed bulk compositions of multiphase fluid inclusions. (A)Ternary diagram showing relative phase proportions of the inclusions, with endmembers defined as brine (H_2O + dissolved NaCl), calcite, and chalcopyrite. (B) Ternary diagram illustrating reconstructed bulk compositions of the inclusions, with endmembers defined as H_2O + NaCl, $CuFeS_2$, and $CaCO_3$. In both diagrams, the data cluster tightly, indicating a high degree of compositional uniformity among the inclusions and supporting a common origin for the trapped fluids.

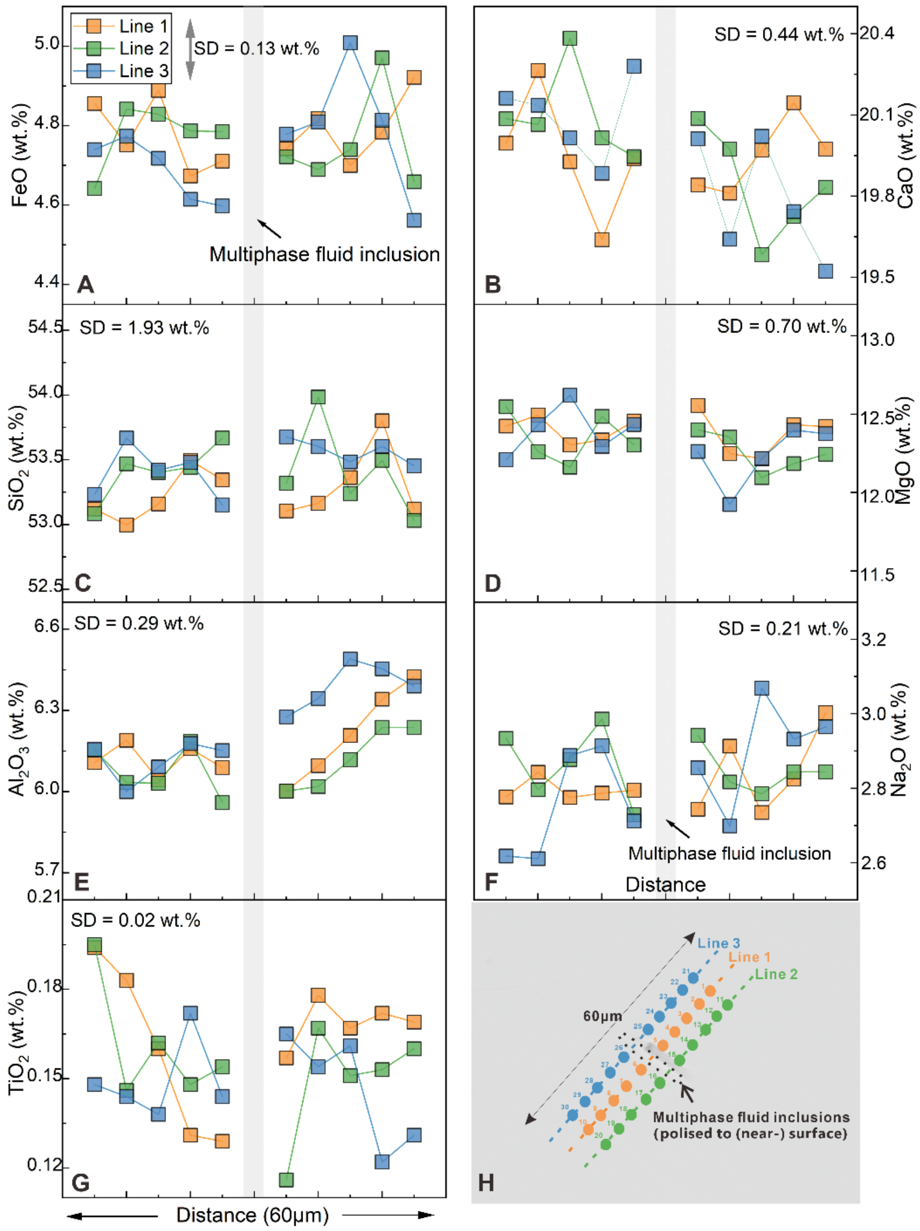

Fig. S6 Major-element variations in host omphacite across a representative multiphase fluid inclusion. (A–G) Electron probe microanalysis (EPMA) profiles showing variations in FeO, CaO, SiO_2, MgO, Al_2O_3, Na_2O, and TiO_2 (wt.%) in host omphacite across a representative multiphase fluid inclusion. Three parallel analytical traverses (Lines 1–3) were conducted across the inclusion, with the grey shaded region marking the position of the fluid inclusion. (H) Schematic illustration showing the relative positions of the three traverses and the fluid inclusion polished to (near-)surface. Overall, major-element compositions of omphacite show only slight variations (e.g., ~0.4 wt.% for FeO and ~0.6 wt.% for CaO) across the inclusion, indicating that post-entrapment modification of the host mineral is minor.

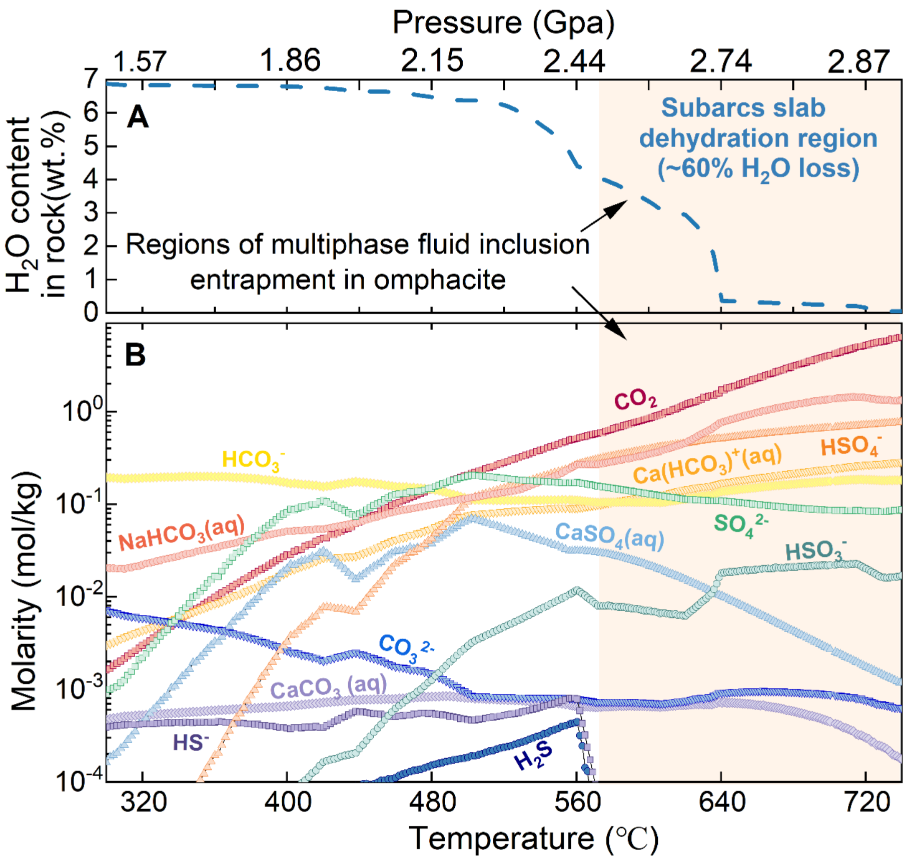

Fig. S7 Modeled slab dehydration and sulfur speciation in subduction zone fluids constrained by the Sumdo system. (A) Modeled evolution of H_2O content in subducted oceanic crust as a function of temperature and pressure, highlighting a pronounced dehydration interval at sub-arc depths (~2.4–2.8 GPa) corresponding to ~60% H_2O loss. (B) Modeled temperature- and temperature-dependent molalities of major sulfur- and carbon-bearing aqueous species in slab-derived fluids, including sulfate (SO_4^{2-}, HSO_4^-), reduced sulfur species (HS^-, H_2S), and carbonate species (HCO_3^-, CO_3^{2-}, $CaCO_3$(aq)), calculated using thermodynamic modeling. Together, these results illustrate efficient slab dehydration at sub-arc depths and the dominance of oxidized sulfur species in slab-derived fluids under the modeled sub-arc conditions. The modelling parameters and results are provided in Supplementary Date 4 and 5.

Supplementary References

1. Liu, H., Xiao, Y., van den Kerkhof, A., Wang, Y., Zeng, L. & Guo, H. Metamorphism and fluid evolution of the Sumdo eclogite, Tibet: constraints from mineral chemistry, fluid inclusions and oxygen isotopes. *J. Asian Earth Sci.* **172**, 292–307 (2019).
