# Supplementary material for: Sulfur-enriched sub-arc fluids drive deep sulfur cycling in subduction zones
Source: Nat Commun. 2026 Apr 7;17:4953. doi: 10.1038/s41467-026-71439-3 (PMC13234425; doi:10.1038/s41467-026-71439-3)
Supplement: Supplementary file 2 — Description of Additional Supplementary Files [file 41467_2026_71439_MOESM2_ESM.pdf]

Description of Additional Supplementary Files

File Name: Supplementary Data 1
Description: Summary table of 3D Raman modeling results for multiphase fluid inclusions in Sumdo eclogites.

File Name: Supplementary Data 2
Description: Bulk chemical compositions (wt.%) of slab-derived fluids at sub-arc depths reconstructed from multiphase fluid inclusions in omphacite.

File Name: Supplementary Data 3
Description: Major-element contents in host omphacite across a representative multiphase fluid inclusion in Sumdo eclogite.

File Name: Supplementary Data 4
Description: Solution model parameters used for thermodynamic modelling.

File Name: Supplementary Data 5
Description: Variations in bulk-rock H_2O and carbon and sulfur concentrations of dissolved species in fluids during rock devolatilization.
